# A comparison of adipose and bone marrow-derived mesenchymal stromal cell secreted factors in the treatment of systemic inflammation

**DOI:** 10.1186/1476-9255-11-1

**Published:** 2014-01-07

**Authors:** Jessica S Elman, Matthew Li, Fangjing Wang, Jeffrey M Gimble, Biju Parekkadan

**Affiliations:** 1 Center for Engineering in Medicine and Surgical Services, Massachusetts General Hospital, Harvard Medical School and Shriners Hospital for Children in Boston, Boston, MA 02114, USA; 2 Harvard-MIT Health Sciences and Technology, Cambridge, MA 02139, USA; 3 Stem Cell Biology Laboratory, Pennington Biomedical Research Center, Louisiana State University, Baton Rouge, LA 70808, USA; 4 Harvard Stem Cell Institute, Boston, MA 02115, USA

**Keywords:** Soluble receptors, Mesenchymal stem cells, Adipose stem cells, Endotoxic shock, Tissue necrosis factor alpha (TNF-α), Vascular endothelial growth factor (VEGF)

## Abstract

**Background:**

Bone marrow-derived mesenchymal stromal cells (BMSCs) are a cell population of intense exploration for therapeutic use in inflammatory diseases. Secreted factors released by BMSCs are responsible for the resolution of inflammation in several pre-clinical models. New studies have uncovered that adipose tissue also serves as a reservoir of multipotent, non-hematopoietic stem cells, termed adipose-derived stromal/stem cells (ASCs), with many common characteristics to BMSCs. We hypothesized that ASC and BMSC secreted factors would lead to a comparable benefit in the context of generalized inflammation.

**Findings:**

Proteomic profiling of conditioned media revealed that BMSCs express significantly higher levels of sVEGFR1 and sTNFR1, two soluble cytokine receptors with known therapeutic activity in sepsis. In a prophylactic study of endotoxin-induced inflammation in mice, we observed that BMSC secreted factors provided a greater survival benefit and tissue protection of endotoxemic mice compared to ASCs. Neutralization of sVEGFR1 and sTNFR1 did not significantly affect the survival benefit experienced by mice treated with BMSC secreted factors.

**Conclusions:**

Our findings suggest that BMSCs may be more effective as a cell therapeutic for use in endotoxic shock and that ASCs may be positioned for continued exploration in immunomodulatory diseases. Soluble cytokine receptors can distinguish stromal cells from different tissue origins, though they may not be the sole contributors to the therapeutic benefit of BMSCs. Furthermore, other secreted factors not discussed in this study may also differentiate these stromal cell populations from one another.

## Introduction

Sepsis is a systemic inflammatory response, typically triggered by bacterial infection, that afflicts 750,000 people each year, more than 210,000 of whom die [1]. The inflammatory response, designed to resolve the infection, affects end-organ perfusion and exacerbates tissue injury leading to multi-organ failure. Sepsis is associated with a surge of systemic signaling molecules including cytokines and growth factors that can become uncontrollable [2]. The body synthesizes naturally occurring soluble cognate receptors for these signaling ligands to regulate these pathways *in vivo*. Soluble receptors are generated by several mechanisms, including proteolytic cleavage of receptor ectodomains, alternative splicing of mRNA, and transcription of genes that encode for the soluble receptor [3]. These synthesized receptors preferentially bind their ligand targets and render them inactive or, conversely, increase their half-life [4]. Their role in the natural progression of sepsis and many other diseases is under investigation [5].

The use of soluble cytokine receptor-based drugs has already been extremely successful on the market. Enbrel® is an engineered version of a soluble tumor necrosis factor alpha-α receptor 1 (sTNFR1) with a longer half-life and is widely prescribed in autoimmune diseases such as rheumatoid arthritis, psoriasis, and Crohn’s Disease [6]. Unfortunately, this treatment has had little success in sepsis [7]. Soluble vascular endothelial growth factor receptor 1 (sVEGFR1) was found to have pre-clinical benefit in a model of bowel perforation leading to bacteremia [8]. A clinical study (NCT01063010) is underway to measure the benefit of reversing the pro-inflammatory, permeability-promoting and procoagulant effects of VEGF at the level of the endothelium based on this finding [9]. Combinatorial approaches to sepsis care that target multiple pathways may be a more effective strategy.

Cell therapy is an alternative drug formulation that uses living cells to achieve a higher-order therapeutic response than a single molecule alone. Bone marrow-derived mesenchymal stem cells (BMSCs) are a connective tissue stem cell population that has been shown to modulate the immune system [10,11]. Transplantation of BMSCs has led to a therapeutic effect in a cecal-ligation sepsis model that was the result of BMSC secreted factors [12]. In a prior study, we reported that human BMSCs secreted 760 pg/10^6^ cells of sTNFR1 [13]. Neutralizing BMSC-derived sTNFR1 partially eliminated the therapeutic benefit to lipopolysaccharide (LPS)-stimulated rats as a model of cytokine response due to gram-negative bacterial infection. Increasing evidence suggests that BMSC secrete molecules that inhibit the effector function of immune cells [14,15], although a more comprehensive view of this immunotherapeutic continues to be sought. Adipose tissue-derived stromal/stem cells (ASCs) are a recently discovered cell population with much in common to their bone marrow-derived counterpart. ASCs are an attractive alternative cell therapy to BMSCs because they can be obtained by less invasive means and are found at a much higher frequency in donor tissue. BMSCs only exist at approximately 0.01% of total nucleated bone marrow cells. ASCs are 500 times more prevalent within an equivalent volume of adipose tissue [16]. Both cell populations have been shown to be effective in certain pre-clinical studies and to mechanistically inhibit the activation of T cells [17]. ASCs and BMSCs have yet to be compared in a model of sepsis and for other anti-inflammatory potency.

In this communication, we present a comparison study of ASCs and BMSCs with a focus on the therapeutic benefit of soluble cytokine receptors in mice suffering from endotoxic shock. BMSCs secrete two soluble cytokine receptors, sTNFR1 and sVEGFR1, in conditioned medium (CM) analysis at dramatically higher levels than ASCs, suggesting a more potent cell population. A prophylactic study in mice subjected to LPS-induced sepsis confirmed a significant survival benefit of BMSC-derived secreted factors compared to ASCs. A subsequent study in LPS-treated mice showed no significant difference in the survival benefit of BMSC-CM compared to conditions in which neutralizing antibodies to sTNFR1 and sVEGFR1 were applied to BMSC-CM. This study indicates that though there are differences in soluble cytokine receptors in secretions of BMSCs compared to ASCs, other factors may play a role in explaining the superior anti-inflammatory effect of BMSCs to ASCs in this model of sepsis.

## Materials and methods

### Mice

BALB/cJ mice (6 weeks) were purchased from The Jackson Laboratory (Bar Harbor, ME) and were maintained in accordance with Institutional and NIH guidelines. Experiments were approved by the Institutional Animal Care and Use Committee of the Center for Comparative Medicine at the Massachusetts General Hospital.

### MSC isolation and culture

Human bone marrow MSCs were isolated by differential adhesion from a 30 mL bone-marrow aspirate obtained from the iliac crest of two human donors (Lonza, Hopkinton, MA). Mononuclear cells (MNC) were isolated from the bone marrow with ACK Lysis Buffer (Lonza), then resuspended in MSC growth medium consisting of Minimum Essential Medium Alpha supplemented with 10% FBS (Gibco, Grand Island, NY), 2% Penicillin-Streptomycin (Sigma Aldrich, St. Louis, MO), 2.5 μg/L FGF (R&D), 2 ml/L Gentamicin (Sigma Aldrich, St. Louis, MO), and 2.2 g/L NaHCO_3_. MNCs were incubated in 10-stack cell factories (Nunclon Delta Surface, Rochester, NY) and allowed to adhere for one week in a humidified tissue culture incubator at 37°C with 10% carbon dioxide. On day 7 of growth, non-adherent cells were removed with full replacement of the growth media. Cells attached to the surface were expanded over another two week period. A PBS rinse and media replacement was performed on day 14. Cells were harvested at day 21 using Trypsin-EDTA (CellGro, Manassas, VA). BMSCs were frozen in cryovials at 1,000,000 cells per vial in 1 mL of cryopreservation medium.

Adipose Stromal/Stem Cell (ASC) Preparation: Human ASC were isolated from donated elective subcutaneous lipoaspirates and abdominoplasty surgeries from two donors under a protocol approved by the Pennington Biomedical Institutional Review Board (PBRC #23040) according to published methods [18,19]. Briefly, lipoaspirate tissues were washed with phosphate buffered saline (PBS), digested for 1 hr in PBS supplemented with 1% bovine serum albumin, 0.1% collagenase type 1 and 2 mM CaCl_2_, and the stromal vascular fraction (SVF) cells isolated by centrifugation at 300 × g at room temperature. The SVF cells were culture expanded in DMEM/F12 Ham’s medium supplemented with 10% fetal bovine serum and 1% antibiotic/antimycotic until >80% confluent. The adherent ASCs were harvested by trypsin digestion, characterized *based surface* immunophenotype as determined by flow cytometry and by adipogenic and osteogenic differentiation prior to cryopreservation in aliquots of 10^6^ cells suitable for future studies.

For expansion of master cell banks, cells were thawed, resuspended in fresh expansion medium, and plated at a density of 50 cells/cm^2^. After 5 days in culture, cells were washed and given fresh media. Within 6–8 days later they were harvested using 0.1% trypsin (Invitrogen, Grand Island, NY) and replated. Cells were used for conditioned media during passages 2–5.

### Preparation of conditioned media (CM)

Cells between passages 2–5 at 80% confluency were washed and MSC media was replaced with 15 ml of conditioning media. Conditioning media consisted of Dulbecco’s Modified Eagle Medium (Invitrogen) supplemented with 2% Penicillin-Streptomycin, .05% BSA (Sigma Aldrich), and 3.6 g/L NaHCO_3._ After 24 hours, media was collected and cells were trypsinized and counted. CM was stored at 4°C for up to two weeks. Concentrated CM was made by centrifuging in Amicon Ultra-15 Centrifugal Filter Units (Millipore, Billerica, MA) repeatedly at 4000 rpm. Concentrated CM was used at a 25-fold concentration of 2×10^6^ cells/ml.

### Survival study in endotoxemia-induced mice

Endotoxemia was induced with intraperitoneal (IP) injections of 10 μg LPS in white female BALB/cJ mice, followed by 1 ml BMSC-CM, ASC-CM, or saline (control) injections IP. There were 9 animals per group. Mice were monitored for survival at several intervals for 48 hours.

### Measurement of sTNFR1, sVEGFR1, sVEGFR2, sTNFR2 in CM

Concentrated CM was subjected to a human sTNFR1/TNFRSF1A DuoSet ELISA Development System (R&D Systems, Minneapolis, MN) according to manufacturer’s protocol. Conditioning media served as the control. We expanded CM analysis for other soluble receptor analytes using a multiplexed Human Soluble Cytokine Receptor Panel Kit (Millipore) per vendor instructions.

### MSC-CM treatment following LPS stimulation in mice for histological studies

Female BALB/cJ mice were given 10 μg LPS IP followed by 1 ml BMSC-CM, ASC-CM, or saline (control) IP. There were four animals per group. Kidney, lung, and liver were harvested from each experimental group, as well as from healthy mice, 24 hours later. Formalin-fixed, paraffin-embedded samples were sectioned and subjected to hematoxylin and eosin (H&E) staining. Magnifications were at ×20. Blind analysis of neutrophil infiltration and scoring of the histological samples were performed by a certified veterinary pathologist. The following criteria were graded: tubular degeneration in kidney, inflammation in the lungs, and hepatocellular vacuolation and degeneration of the liver.

### Antibody neutralization of soluble cytokine receptors in BMSC-CM

To induce endotoxemia, female BALB/cJ mice were given 10 μg LPS IP. Mice were then given 1 mL BMSC-CM, 1 mL BMSC-CM and neutralizing sTNFR1 antibody, 1 mL BMSC-CM and neutralizing sVEGFR1 antibody, or conditioning media not exposed to cells, which served as a control. Dosage of neutralizing antibodies to sTNFR1 and sVEGFR1 (R&D Systems) was calculated based on the vendor’s instructions to apply 1000–6000 ng/ml antibody in the presence of 300 ng/ml of soluble cytokine receptor. Based on our previous measurements, there were 1.5 ng/ml of sTNFR1 in concentrated BMSC-CM. Thus, we applied 25 ng/ml neutralizing sTNFR1 antibody to BMSC-CM to bind and block most of the sTNFR1. There were approximately 2 ng/ml of sVEGFR1 in BMSC-CM, we applied 36 ng/ml of neutralizing sVEGFR1 antibody to BMSC-CM. Mice were monitored for survival for 48 hours. This study was independently repeated with the same treatment groups to arrive at the final cohort numbers.

### Statistical analysis

ELISA results were analyzed using an unpaired Student’s t-test assuming a normal distribution. All data represents the mean of the samples ± standard deviation. For survival study analysis, a log rank test was performed. P < 0.05 values were considered statistically significant.

## Results

### Significantly increased secretion of sTNFR1 and sVEGFR1 in BMSC-CM versus ASC-CM

**Figure 1:**
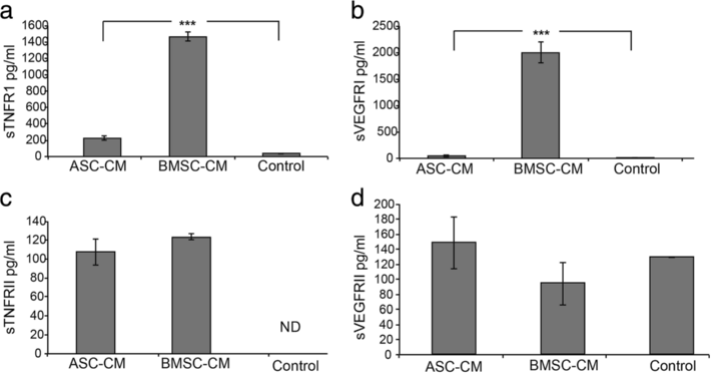
**Differences in soluble receptor secretion by ex-vivo expanded stromal cell populations.** BMSC-CM and ASC-CM was concentrated 25-fold, to a formulation that represented the collection of 2x10^6^ cells per ml of CM. Control samples are basal media that was not conditioned by cells. Shown are ELISA analysis of CM for soluble receptors, including **(a)** sTNFR1, **(b)** sVEGFR1, **(c)** sTNFR2 and **(d)** sVEGFR2. There was non-specificity of the sVEGFR2 antibody pair when analyzing CM samples. BMSCs uniquely expressed high levels of sTNFR1 and sVEGFR1.

We began our study by measuring the presence of sTNFR1 from human BMSCs in comparison to ASCs derived from a lipoaspirate. Multiplex ELISA analysis of concentrated CM (2×10^6^ cells conditioned/ml of CM) was used to detect soluble cytokine receptors specifically related to the TNF-α and VEGF pathways. Stark differences in sTNFR1 and sVEGFR1 quantities were observed between the two cell populations. BMSCs secrete over six times as much sTNFR1 as ASCs (Figure 1a; BMSC: 731.6 ± 30 pg/10^6^ cells, ASC: 116.5 ± 13 pg/10^6^ cells, P = 5.05 × 10^-6^). ASCs had nearly undetectable levels of sVEGFR1 (20.5 ± 7.8 pg/10^6^ cells). BMSCs secreted 1000.85 pg/10^6^ cells of sVEGFR1 under the same conditions (Figure 1b; P = 7.5×10^-5^). Other soluble cytokine receptors (Figure 1c-d) did not differ significantly. These data led to the hypothesis that BMSC secreted factors would be more potent in a model of sepsis than ASC factors.

### BMSC-CM provides superior survival benefit compared to ASC-CM in the prevention of endotoxic shock in mice

**Figure 2:**
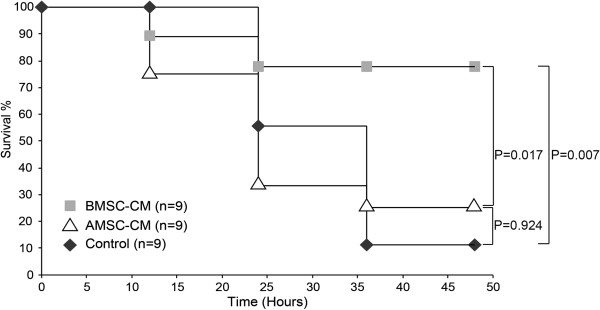
**BMSC-CM provides a greater survival benefit to LPS-treated mice than ASC-CM.** 10 ug LPS were administered IP to each mouse to induce endotoxemia, followed by either 1 ml of saline (control), 1 ml BMSC-CM, or 1 ml ASC-CM IP. ASC-CM treated mice did not experience a significantly greater survival benefit in comparison to control (P = 0.924), whereas BMSC-CM treated mice did (P = 0.007).

We exposed mice to a supraphysiological level of LPS as a model of sepsis. In response to LPS stimulation, a subject’s immune system overwhelms the body with an exaggerated cytokine response. We evaluated the potential of CM derived from BMSCs to ASCs to prevent the sequelae caused by this “cytokine storm” *in vivo*. Mice received a single intraperitoneal injection of LPS (10 μg per 25 g mouse), followed by 1 ml intraperitoneal injection of concentrated BMSC-CM or ASC-CM (25-fold; 2×10^6^ cells conditioned/ml). This concentration was used based on previous findings reported that this concentration regimen can induce a therapeutic response in rodents [14,20]. As Figure 2 shows, mice that received either saline or ASC-CM displayed significantly higher levels of mortality than the BMSC-CM treated group. By the end of the study, 78% of the BMSC-CM animals were alive and recovering, whereas only 25% of the ASC-CM and 11% of the control animals survived (P = 0.007 of BMSC-CM vs. saline; P = 0.017 of BMSC-CM vs ASC-CM; P = 0.924 of ASC-CM vs. saline).

### BMSC-CM provides greater multi-organ protection in LPS induced injury compared to ASC-CM

**Figure 3:**
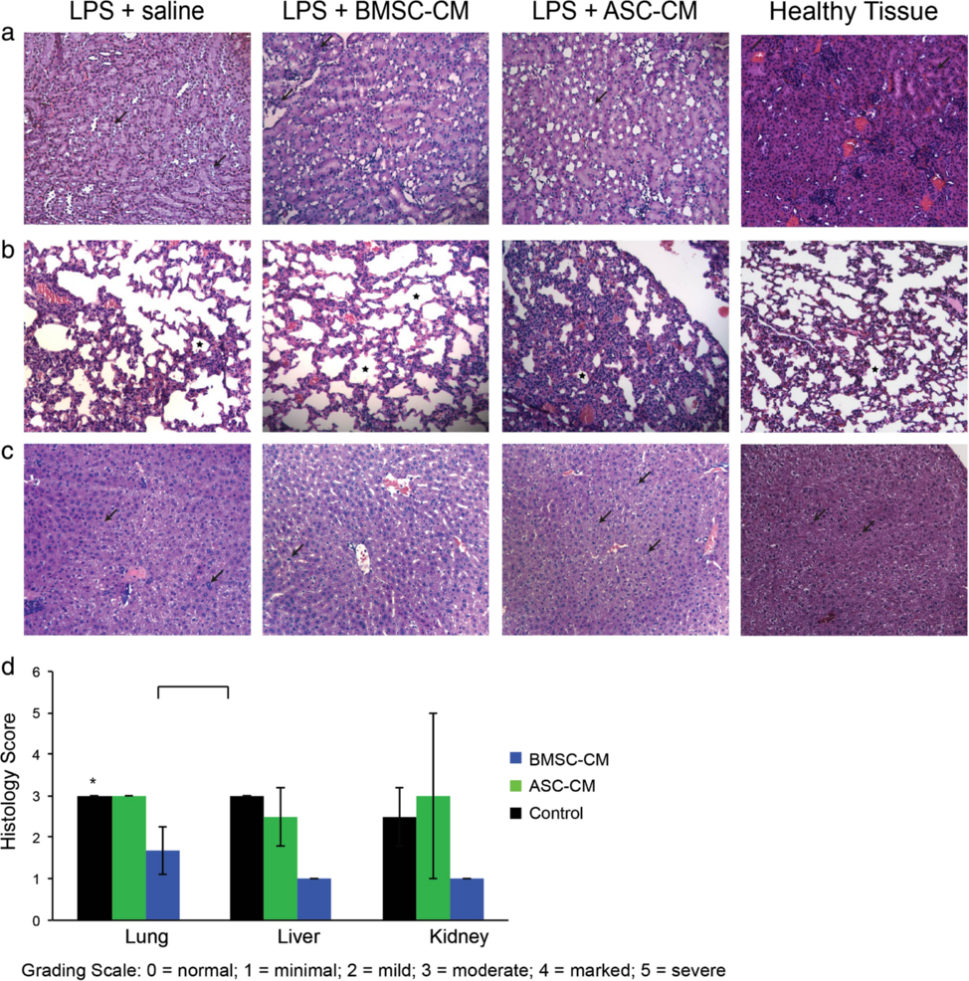
**Cell-specific attenuation of end-organ damage in LPS mice by BMSC-CM.** One day following LPS and CM administration, tissue was harvested for hematoxylin and eosin (H&E) staining. Sections were analyzed for detection of tubular degeneration in kidney, inflammation in the lungs, hepatocellular vacuolation and degeneration of the liver. Magnifications are x20. **(a)** The renal cells of the healthy kidney appear to be unperturbed in contrast with the pale and poorly defined cells of the LPS-treated mouse. The ASC-CM kidney presents with similarly pale renal cells, whereas the BMSC-CM kidney resembles the healthy control. **(b)** Alveoli are generously and evenly distributed in the healthy lung compared to the LPS-treated group, which appears more constricted. The BMSC-CM treated lungs also present with expansive and unaffected alveoli, but the ASC-CM treated lungs are clearly devastated: alveoli are significantly smaller and sparser. **(c)** The healthy liver shows few signs of inflammation. The LPS-treated liver has swollen hepatocytes with affected nuclei. Many unhealthy cells appear in the ASC-CM treated liver as well. The BMSC-CM treated liver appears to have fewer necrotic hepatocytes. **(d)** Histological slides were blindly scored by a pathologist. (P < 0.05).

The infiltration of neutrophils and macrophages into different organs is a characteristic marker of the general inflammation caused by endotoxic shock [21]. To visualize the degree of protection to endotoxic organs, we harvested the kidney, lungs, and livers from mice exposed to LPS and treated with BMSC-CM and ASC-CM. Histological analysis was performed one day after treatment to ensure a high level of survival in all groups. Kidneys treated with BMSC-CM exhibited the least neutrophilic infiltration and the greatest preservation in tubule architecture (Figure 3a). The BMSC-CM lungs (Figure 3b) also had unperturbed alveoli, unlike the control or ASC-CM groups. Liver histology (Figure 3c) also revealed increased vacuolation and degeneration in control and ASC-CM groups, in contrast to the BMSC-CM-treated group. A blinded pathological score for these tissues quantified the observations that BMSC-CM treated mice collectively had the least amount of end-organ damage (Figure 3d) and were significantly different than ASC-CM treated mice (P < 0.05).

### Neutralization of sTNFR1 and sVEGFR1 does not statistically inhibit preventative effects of BMSC-CM on endotoxic shock in mice

**Figure 4:**
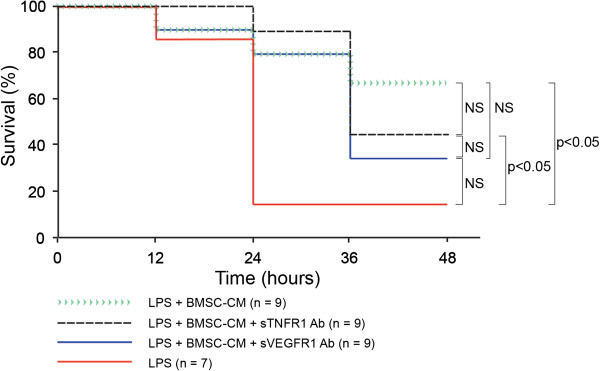
**LPS-treated mice experience insignificant benefit when treated with BMSC-CM compared to BMSC-CM with neutralizing antibodies targeting sTNFR1 and sVEGFR1.** 10 ug LPS were administered IP to each mouse to induce endotoxemia, followed by either 1 ml of saline (control), 1 ml BMSC-CM, 1 ml BMSC-CM with neutralizing sTNFR1 antibody, or 1 ml BMSC-CM with neutralizing sVEGFR1 antibody IP. BMSC-CM treated mice experienced a significantly greater survival benefit in comparison to control mice (P = 0.0454). Mice given BMSC-CM with neutralizing sTNFR1 antibody also experienced a significantly greater survival benefit compared to control (P = 0.0225). The BMSC-CM with neutralizing sVEGFR1 antibody group did not differ significantly from the control (P = 0.106) or from the neutralizing sTNFR1 antibody group (P = 0.528). Finally, neither group that received a neutralizing soluble receptor antibody had a significantly different survival benefit compared to the BMSC-CM treated group (neutralizing sTNFR1 antibody vs BMSC-CM P = 0.563; neutralizing sVEGFR1 antibody vs BMSC-CM P = 0.237).

Neutralization of sTNFR1 and sVEGFR1 in BMSC-CM was tested to determine if survival was caused by these soluble cytokine receptors *in vivo*. In order to minimize changes in the overall composition of CM, we used neutralizing antibodies rather than engineering of cells with genetic constructs that could potentially induce new and possibly unknown factors. Mice received an intraperitoneal injection of LPS (10 μg per 25 g mouse) followed by a 1 ml intraperitoneal injection of concentrated BMSC-CM (25-fold; 2×10^6^ cells conditioned/ml), BMSC-CM treated with neutralizing sTNFR1 antibody (0.025 ug/ml), or BMSC-CM treated with neutralizing sVEGFR1 antibody (0.036 ng/ml). We found that the survival benefit from BMSC-CM was generally unaffected by the neutralization of sTNFR1 and sVEGFR1. As Figure 4 shows, survival benefit insignificantly differs between the groups that received the neutralizing antibodies and the regular BMSC-CM. By the end of the 48-hour study, 66% of the BMSC-CM, 44% of the neutralizing sTNFR1 antibody-treated BMSC-CM, and 33% of the neutralizing sVEGFR1 antibody-treated BMSC-CM mice were alive and recovering. Of the LPS-treated control mice, only 14% survived. This group differed significantly from the neutralizing sTNFR1 antibody BMSC-CM group (P < 0.05) and from the BMSC-CM group (P < 0.05). These data suggest that factors other than soluble cytokine receptors may contribute to the survival benefit BMSC-CM gives to mice suffering from endotoxic shock.

## Discussion

ASCs and BMSCs display similar morphology and growth characteristics when expanded *ex vivo*. Immunophenotypically, they are both known to be positive for the classical mesenchymal stromal cell markers CD105, CD44 CD90, CD166, and negative for CD14, CD19, and CD45 [16]. There is a continued effort to understand the differences between these MSCs, particularly as they are both being advanced for cell immunotherapeutic purposes. ASCs and BMSCs have comparable suppressive effects on the growth of PHA-stimulated T cells, suggesting that they have equal influence on the adaptive immune response [9]. We explored their comparability in preventing innate immune responses observed in endotoxic inflammation and demonstrate a significant improvement of disease indices by BMSC secreted factors. We discovered that BMSCs secrete significantly greater amounts of two soluble cytokine receptors, sTNFR1 and sVEGFR1, compared to ASCs. Although these soluble cytokine receptors can be considered potency markers unique to BMSCs, other factors are likely to also be responsible for BMSCs’ therapeutic effects in systemic inflammatory responses.

sTNFR1 has been shown to block the effects of TNF-α, an inflammatory cytokine that is released in response to inflammation. With increased distress, TNF-α reaches dangerously high levels and can eventually lead to death [22]. TNF-α converting enzyme (TACE) has been implicated as the enzyme that sheds sTNFR1 from TNFR1. Upon sTNFR1’s release, it binds to circulating TNF-α and prevents its inflammatory effects [11]. A study by Yagi *et al*. demonstrated the value of circulating sTNFR1 released by intramuscular BMSC transplants in the attenuation of septic shock in rodents. When BMSC transplants were co-administered with a neutralizing sTNFR1 antibody, the cell therapy failed to prevent the infiltration of inflammatory cells in the lungs, liver, and kidney [6]. The downstream mechanism by which sTNFR1 is protective remains to be determined. There is evidence that sTNFR1 may not just simply block TNF-α, but exert its effects by inducing apoptosis in monocytes via transmembrane TNF-α [12]. Waetzig *et al.* found that sTNFR1 boosts TGFβ1, which in turn has the ability to inhibit T lymphocyte proliferation [13]. There is, however, a delicate balance of the sTNFR1/TNF-α axis where it has been observed that low sTNFR1 levels stabilize TNF-α [23]. Further studies will be needed to unravel the mechanism that BMSCs employ to make sTNFR1 and how this soluble receptor modulates the immune system.

VEGF, an angiogenic factor that supports microcapillary growth from existing vasculature, typically benefits endothelial tissues [14]. Recent studies have shown that elevated levels of VEGF in response to inflammation can cause more hindrance than initially thought. A study by Tsao *et al.* demonstrated that injecting mice with VEGF after induction of endotoxemia via LPS resulted in 100% mortality [8]. VEGF induces the expression of cell adhesion molecules, which cause leukocytes to bind effectively to the endothelium. This can lead to even greater local cytokine production. VEGF can be silenced by sVEGFR1, the truncated form of membrane-bound VEGF receptor 1 [15]. Heightened sVEGFR1 levels can mitigate this devastating cascade at the blood vessel scale. Mice treated with sVEGFR1 as late as 20 hours after LPS administration have demonstrated a survival rate of 100% [16]. Several theories have been proposed to explain the dichotomous nature of an anti-angiogenic factor, sVEGFR1, as a beneficial influence in inflammatory disease. Some suggest that high levels of sVEGFR1 in sepsis may promote hypocoagulability by recruiting endogenous anti-coagulating molecules, which counteract the tissue damage by allowing microcirculation to remain open and permit the body to recover more quickly from endotoxemia [15]. sVEGFR1 has also been shown to selectively activate endothelial nitric oxide synthase (eNOS), which contributes to arteriogenesis, angiogenesis, and mural cell recruitment [17]. Apart from sepsis, the presence of sVEGFR1 is also thought to regulate angiogenesis by VEGF in the setting of cancerous tumor growth [24]. The release of sVEGR1 by BMSCs may have implications in the therapeutic use of this cell population as well as the endogenous regulation of VEGF signaling in stromalized microenvironments.

Our findings indicate, however, that modulating sVEGFR1 activity alone with a neutralizing antibody does not significantly affect BMSC-CM’s benefit to sepsis survival. For the studies utilizing neutralizing antibodies, the addition of IgG to the BMSC-CM would ideally be used to control for the presence of antibody in the neutralizing antibody-treated CM. Its absence, however, is likely to be inconsequential because the bovine serum albumin IgG in the CM acts as a sufficient control for the neutralizing antibody’s influence on the LPS-challenged mice.

In conclusion, we have uncovered a potency advantage of BMSCs compared to ASCs in a model of sepsis and a unique expression pattern of soluble receptors that are implicated in the resolution of inflammation. This study can guide the positioning and monitoring of these cell populations for therapeutic use in immune-mediated disease and may have ramifications to endogenous stromal cell biology.

## Competing interests

Dr. Gimble is the co-founder, co-owner and Chief Scientific Officer at LaCell LLC, a for-profit biotechnology company focusing on stromal/stem cell related products isolated from discarded medical waste tissues, including adipose and bone marrow.

## Authors’ contributions

JE: conceived and performed experiments, analyzed data, and wrote the manuscript. ML: conceived and performed survival study experiments. FW: helped perform *in vivo* experiments. JG: contributed ASCs. BP: conceived and performed experiments, and helped write the manuscript. All authors read and approved the final manuscript.
